# High frequency of enterotoxin encoding genes of *Staphylococcus aureus* isolated from food and clinical samples

**DOI:** 10.1186/s41043-021-00246-x

**Published:** 2021-06-09

**Authors:** Fakhri Haghi, Habib Zeighami, Zeynab Hajiloo, Neda Torabi, Safoura Derakhshan

**Affiliations:** 1 Department of Microbiology, School of Medicine, Zanjan University of Medical Sciences, Zanjan, Iran; 2 Student Research Committee, Zanjan University of Medical Sciences, Zanjan, Iran; 3 Department of Microbiology, Zanjan Branch, Islamic Azad University, Zanjan, Iran; 4 Department of Microbiology, Faculty of Medicine, Kurdistan University of Medical Sciences, Sanandaj, Iran

**Keywords:** *Staphylococcus aureus*, Classical enterotoxins, Clinical samples, Meat, PCR

## Abstract

**Background:**

*Staphylococcus aureus* is recognized as an important cause of food poisoning related to the consumption of raw, undercooked, or mishandled foods worldwide.

**Methods:**

A total of 90 individual meat samples and 200 clinical specimens were collected and investigated the frequency of *S. aureus* and classical enterotoxin genes. The samples were cultured on Baird-Parker and Mannitol salt agar and subjected for confirmatory biochemical tests and molecular detection of *femA*, *sea*, *seb*, *sec*, *sed*, and *see* genes.

**Results:**

A total of 31 (34.5%) meat samples and 81 (40.5%) clinical specimens were positive for the presence of *S. aureus.* These isolates were detected with slightly higher frequency in clinical specimens than food samples (*P*> 0.05). Furthermore, the frequency of *S. aureus* in raw meat (23.4%) was higher than that in cooked meat samples (11.1%) (*P*< 0.05). Staphylococcal enterotoxin (SE) genes were identified in 18 (58.1%) of 31 meat isolates and 42 (51.8%) of 81 clinical isolates. The frequency of SE genes (except *see*) in meat isolates was slightly higher than that in clinical isolates (*P*> 0.05). We found *sea* and *see* genes with higher frequency than others in both meat and clinical samples. Furthermore, 55.5% of meat isolates and 38.1% of clinical isolates possessed more than one *se* gene.

**Conclusion:**

Detection of enterotoxigenic *S. aureus* in clinical and raw meat samples shows a probable risk for public health. Therefore, intensive and continuous monitoring of potentially pathogenic *S. aureus* is strongly recommended in order to evaluate the human health risk arising from food consumption.

## Background

*Staphylococcus aureus* is one of the most common causes of bacterial food poisoning worldwide, causing an estimated 241,148 cases and 6 deaths per year in the USA [1–3]. According to a report of Iranian centers of public health in the central province of Iran, the prevalence of staphylococcal food poisoning in this region is considerable [4]. Staphylococcal food poisoning (SFP) is related to the consumption of foods containing sufficient amounts of one or more preformed enterotoxins [5–8]. The high incidence of staphylococcal food poisoning is due to the insufficient pasteurization/decontamination of originally contaminated product source or its contamination during preparation and handling by individuals who are carriers of the organism [9].

Because the skin and mucous membranes of animals are considered as the reservoirs for *S. aureus*, this microorganism has enough potential to exist and transmit to or contaminate and spoil animal products such as milk and meat [10]. Foods that have been frequently incriminated in staphylococcal intoxication include raw retail meat and meat products, poultry and egg products, milk and dairy products, salads, bakery products, particularly cream-filled pastries and cakes, and sandwich fillings [11]. As *S. aureus* is frequently isolated in raw milk, it is responsible for an increase in milk somatic cell count and a decrease in milk’s nutritional composition and productivity [12].

*S. aureus* produces a wide variety of toxins belonging to the fascinating family of superantigens including staphylococcal enterotoxins (SEs; SEA-SEE, SEG-SEI, and SER-SET) and staphylococcal enterotoxin-like (SEls) toxins, which their emetic properties remain unconfirmed (SElK-SElQ and SElU-SElX). SEs and SEls are single-chain proteins with size range from 22 to 29 KDa and encoded by accessory genetic elements including plasmids, prophages, pathogenicity islands, and genomic island vSa, or by genes located next to the staphylococcal cassette chromosome (SCC) [11, 13–15]. The amount of SE required for the establishment of typical symptoms of food poisoning including nausea, vomiting, emesis, stomach cramps, and diarrhea is very low and approximately ranging from 20 ng to 1 μg [16–18]. Among SEs, SEA is the most common cause of staphylococcal food poisoning worldwide, but the involvement of other classical SEs has been also demonstrated [11]. Due to the stability of SEs in denaturing conditions such as heat and low pH, these toxins are not completely destroyed by mild cooking or digestion of food in the stomach [19, 20].

Although enterotoxigenic staphylococci are thermally destroyed, the cooked meat products may contain SEs because these toxins are thermostable and cannot be destroyed by heat processing. This fact represents a serious hazard to healthy consumer when ready-to-eat meat products are processed [21]. Therefore, it is essential to detect SE-producing staphylococci and gather information about other microbial risk factors and hazards associated with raw and pre-processed meat products. Risk assessment and microbial monitoring will continue to play important role in the quality assurance of meat products [21].

The detection of *S. aureus* and SEs in food is difficult. Methods currently used for the detection of SEs in food are enzyme-linked immunosorbent assay (ELISA), reversed passive latex agglutination (SET-RPLA), and polymerase chain reaction (PCR) technique [19].

Several studies have investigated staphylococcal enterotoxins in food samples such as raw milk and dairy products, but there are few such studies on meat samples [10]. Furthermore, there is a lack of information on the occurrence of enterotoxin producing *S. aureus* in meat and clinical samples in our region. So, we aimed in the present study to investigate the presence and the frequency of *S. aureus* and classical SE genes in meat and clinical samples in Zanjan, Iran.

## Methods

### Patients and sampling

This prospective study was approved by the Research Ethics Committee of Zanjan University of Medical Sciences (IR.ZUMS.REC.1394.69) and informed written consent was obtained from patients. The study population was comprised of all hospitalized patients with clinical presentations such as urinary tract infection, bacteremia, meningitis, synovitis, and wound infections admitted to four major university hospitals in Zanjan, Iran, from March to June 2015. Patients were enrolled in the study if they had not taken any antimicrobial agent in the week preceding sampling. Clinical specimens including blood (47), synovial fluid (22), urine (29), CSF (33), and wound (69) were collected from patients. One sample from each patient was collected and transported to the laboratory of Medical Microbiology in a cool box within 1 h.

Due to the distribution of meat retail outlets and restaurants in Zanjan, the cluster sampling method was used. At first, Zanjan was divided into 4 geographical regions based on the abundance of meat retail outlets and restaurants. Then, a total of 19 restaurants and 25 meat retail outlets were selected. One to three samples were collected from each meat retail outlet and restaurant.

From March to June 2015, a total of 90 individual meat samples including raw beef (23), raw lamb (22), and cocked meat (45) samples were collected from meat retail outlets and restaurants. Meat samples were packed into a clean polyethylene bag then marked and transported to the laboratory of Microbiology in a cool box for analysis within 1 h.

### Reference strains

Reference strains of *S. aureus* ATCC 13565 (SEA), *S. aureus* ATCC 14458 (SEB), *S. aureus* ATCC 19095 (SEC), *S. aureus* ATCC 23235 (SED), and *S. aureus* ATCC 27664 (SEE) were used as positive controls in this study.

### Isolation and identification of *S. aureus*

Twenty-five grams of meat samples was homogenized for 90 s in a stomacher (Heidolph, Schwabach, Germany) with 225 ml of peptone water (PW) containing 6.5% NaCl and then incubated at 37°C for 24 h. After primary enrichment, a loopful (without shaking the flask) from each of the enriched homogenates was streaked onto Baird-Parker agar (MERCK, Darmstadt, Germany) supplemented with 5% egg yolk and tellurite and incubated under aerobic conditions at 37 °C for 24 h. Colonies with typical gray-black appearance surrounded by a clear zone were enumerated as coagulase-positive staphylococci and sub-cultured onto Mannitol salt agar*.* Clinical specimens were also cultured onto Brain Heart Infusion agar and Mannitol salt agar (MERCK, Darmstadt, Germany) [22, 23]. The isolates were identified as *S. aureus* by further biochemical characterization using Gram stain, catalase, coagulase, oxidase, lipase, DNase, and PCR targeting the *S. aureus*-specific *femA* gene (*S. aureus* species specific).

### Genomic DNA extraction

A colony of *S. aureus* (one colony per sample) was picked from nutrient agar and inoculated into 5 ml of LB (Luria Bertani Broth, Merck) until the exponential phase with 2 McFarland turbidity with shaking at 120 rpm at 37 °C. Extraction of genomic DNA was performed according to the protocol provided with the Qiagen Mini Amp kit.

### Detection of *sea*-*see* in *S. aureus* isolates by PCR

The presence of staphylococcal enterotoxin genes *sea*, *seb*, *sec*, *sed*, and *see* was assessed using the primers listed in Table 1. Single PCR was performed using DreamTaq PCR Master Mix (Thermo Fisher Scientific), which contains Taq polymerase, dNTPs, MgCl_2_, and the appropriate buffer. Each PCR tube contained 25 μl reaction mixture composed of 12.5 μl of the master mix, 2.5 μl of each forward and reverse primer solution (in a final concentration of 200 nM), 2 μl of DNA with a concentration of 400 ng, and nuclease-free water to complete the final volume. PCR was performed using the Gene Atlas 322 system (ASTEC) with the same cycling conditions for *sea-see* genes. Amplification involved an initial denaturation at 94°C, 5 min followed by 30 cycles of denaturation (94°C, 1.5 min), annealing (55 °C, 1.5 min), and extension (72 °C, 1.5 min), with a final extension step (72 °C, 8 min). The amplified DNA was separated by submarine gel electrophoresis on 1.5% agarose, stained with ethidium bromide, and visualized under UV transillumination.

**Table 1:** Primers used in this study

Target	Primer sequence (5′→3′)	Amplicon size (bp)	References
***femA***	**AAAAAAGCACATAACAAGCG** **GATAAAGAAGAAACCAGCAG**	**132**	[24]
***sea***	**CCTTTGGAAACGGTTAAAACG** **TCTGAACCTTCCCATCAAAAAC**	**127**	[25]
***seb***	**TCGCATCAAACTGACAAACG** **GCAGGTACTCTATAAGTGCC**	**477**	[25]
***sec***	**CTCAAGAACTAGACATAAAAGCTAGG** **TCAAAATCGGATTAACATTATCC**	**271**	[25]
***sed***	**CTAGTTTGGTAATATCTCCTTTAAACG** **TTAATGCTATATCTTATAGGGTAAACATC**	**319**	[25]
***see***	**CAGTACCTATAGATAAAGTTAAAACAAGC ** **TAACTTACCGTGGACCCTTC**	**178**	[25]

### Statistical analysis

The data were analyzed with SSPS version 17.0 software (SPSS, Inc., Chicago, IL). The chi-square test was used to determine the statistical significance of the data. A *P* value of < 0.05 was considered significant.

## Results

### Frequency of *S. aureus* in clinical samples and meat

Of the total number of patients (200), 38 (19%) were 15–30 years, 72 (36%) were 30–45 years, 58 (29%) were 45–60 years, and 32 (16%) were >60 years. The sex distribution was 55% male and 45% female.

Overall, 81 (40.5%) *S. aureus* isolates were identified in the 200 clinical samples: 20 (10%) isolates of blood, 17 (8.5%) isolates of CSF, 5 (2.5%) isolates of synovial fluid, 29 (14.5%) isolates of wound, and 10 (5%) isolates of urine samples.

A total of 90 individual meat samples were studied for the presence of *S. aureus*. A conventional cultural method based on the appearance of gray-black colonies surrounded by a clear zone on Baird-Parker agar plates was detected coagulase-positive staphylococci in 43 (47.8%) out of the 90 samples. However, the biochemical tests and molecular analysis of *femA* in coagulase-positive staphylococci indicated that 34.5% (31/90) of samples were positive for *S. aureus*: 12 (13.4%) isolates from raw lamb, 9 (10%) isolates from raw beef, and 10 (11.1%) isolates from cooked meat samples (Table 2).

**Table 2:** Frequency of *S. aureus* in meat and clinical samples

Samples (no.)	No. (%) of samples containing ***S. aureus***		
	Positive samples	No.	(%)
Raw lamb (22)	A2, A3, A5, A7, A8, A9, A10, A14, A16, A18, A19, A21	12	13.4
Raw beef (23)	B1, B2, B3, B6, B8, B9, B10, B12, B13	9	10
Cooked meat (45)	C5, C13, C16, C24, C26, C32, C34, C35, C39, C45	10	11.1
Blood (47)	D1, D5, D8-D10, D15, D17, D20, D21, D24, D25-D32, D39, D42	20	10
CSF (33)	E3, E8, E11, E13, E14- E18, E21, E23-E27, E30, E33	17	8.5
Synovial fluid (22)	F6, F12, F17, F20, F22	5	2.5
Wound (69)	G2, G7-G10, G13, G18, G20-G26, G31, G37-G41, G45, G49-G52, G55, G57, G59, G61	29	14.5
Urine (29)	H3-H5, H11, H14, H16, H19, H21, H25, H28	10	5
**Total (290)**	–	**112**	**38.6%**

### Distribution of enterotoxin genes in *S. aureus* isolates

Overall, 58.1% (18/31) of meat isolates were positive for the presence of at least one or more SE genes: 10 isolates (32.3%) from lamb and 8 isolates (25.8%) from beef samples. The frequency of each SE gene in *S. aureus* isolates is shown in Table 3. Comparison of SE gene frequency among beef and lamb isolates showed a different distribution of these genes. The most prevalent SE gene among beef and lamb isolates was *sea* (38.7%), followed by *see* (22.6%), *sec* (16.1%), and *seb* (12.9%). SE genes were not found in strains isolated from cooked meat samples.

**Table 3:** Frequency of SE genes among *S. aureus* isolates

Enterotoxin	No. (%) of meat isolates carrying SE genes (***n***= 18)				No. (%) of clinical isolates carrying SE genes (***n***= 42)					
	Raw lamb isolates (*n*= 12)	Raw beef isolates (*n*= 9)	Cooked meat isolates (*n*= 10)	Total (*n*=31)	Blood isolates (*n*= 20)	CSF isolates (*n*= 17)	Synovial fluid isolates (*n*= 5)	Wound isolates (*n*= 29)	Urine isolates (*n*= 10)	Total (*n*= 81)
***sea***	5 (16.1)	7 (22.6)	0	12 (38.7)	7 (8.6)	5 (6.2)	2 (2.4)	5 (6.2)	4 (4.9)	23 (28.4)
***seb***	3 (9.7)	1 (3.2)	0	4 (12.9)	2 (2.4)	1 (1.2)	1 (1.2)	3 (3.7)	2 (2.4)	9 (11.1)
***sec***	4 (12.9 )	1 (3.2)	0	5 (16.1)	3 (3.7)	2 (2.4)	1 (1.2)	2 (2.4)	0	8 (9.9)
***sed***	1 (3.2)	1 (3.2)	0	2 (6.4)	1 (1.2)	1 (1.2)	1 (1.2)	0	0	3 (3.7)
***see***	3 (9.7)	4 (12.9)	0	7 (22.6)	6 (7.4)	7 (8.6)	2 (2.4)	4 (4.9)	3 (3.7)	22 (27.1)

Among 81 clinical isolates, 42 (51.8%) isolates carried at least one or more enterotoxin genes. The frequency of *sea*, *seb*, *sec*, *sed*, and *see* genes in clinical isolates was 28.4%, 11.1%, 9.9%, 3.7%, and 27.1%, respectively (Table 3).

The presence of multiple SE genes with different combinations was found among isolates. Of 18 meat isolates and 42 clinical isolates harboring enterotoxin genes, 10 (55.5%) and 16 (38.1%) isolates, respectively, had two or more SE genes simultaneously. The number of SEs per isolate and their specific combinations are shown in Table 4. The frequent combination of SE genes in meat isolates was *sea+see* (16.7%), followed by *sea+seb+see* (11.1%). One isolate (5.5%) of lamb samples carried *sea+seb+sec+see* simultaneously. Many of the clinical isolates (61.9%) had only one SE gene (30.9% *sea*, 26.2% *see*, 2.4% *sec*, and 2.4% *sed*). The frequent combination of SE genes among clinical isolates was *sea+seb+see* (9.5%) and *sea+seb* (7.1%), respectively.

**Table 4:** Specific SE combinations among the 26 *S. aureus* isolates (10 meat and 16 clinical isolates) carrying more than one SE genes

SE combinations	No. (%) of isolates carrying SE genes combinations		
	Meat isolates carrying SE genes (*n*=18)	Clinical isolates carrying SE genes (*n*= 42)	Total (*n*=60)
***sea+sed***	1 (5.5)	–	1 (1.6)
***sea+seb***	1 (5.5)	3 (7.1)	4 (6.6)
***seb+sec***	–	1 (2.4)	1 (1.6)
***sec+see***	–	2 (4.7)	2 (2.4)
***sea+sec***	–	1 (2.4)	1 (1.6)
***sea+see***	3 (16.7)	2 (4.7)	5 (6.2)
***sec+sed***	1 (5.5)	1 (2.4)	2 (2.4)
***sea+seb+see***	2 (11.1)	4 (9.5)	6 (10)
***sea+sec+see***	1 (5.5)	–	1 (1.6)
***sea+seb+sec***	–	1 (2.4)	1 (1.6)
***sea+sec+sed+see***	–	1 (2.4)	1 (1.6)
***sea+seb+sec+see***	1 (5.5)	–	1 (1.6)
**Total**	10 (55.5)	16 (38.1)	26 (43.3)

## Discussion

*Staphylococcus aureus* is the most common foodborne pathogen and represents a major public health problem in developing countries [1]. Several studies have shown an increasing prevalence of *S. aureus* in food samples such as raw milk and dairy products, raw meat, and meat products [10, 11, 19]. In our study, a total of 81 (40.5%) clinical specimens and 31 (34.5%) meat samples were positive for the presence of *S. aureus.* These isolates were detected with slightly higher frequency in clinical specimens than food samples (*P*> 0.05). Furthermore, the frequency of *S. aureus* in raw meat (23.4%) was higher than that in cooked meat samples (11.1%) (*P*< 0.05). Only a few reports on the frequency of *S. aureus* in clinical and meat samples from Iran have been published. According to the previous reports from Iran, 3.7–15.6% of the meat samples [26] and 20.8% of clinical specimens [27] were positive for the presence of this pathogen. Raw meat contamination with *S. aureus* has been reported 24% in Italy yielding positive cultures [28]. According to Moon et al. [29] and Aydin et al. [30], the frequency of *S. aureus* in meat products was 36% and 13.8%, respectively.

This variation in *S. aureus* frequency may be due to differences in the geographical region, reservoir in the various countries, sample type, number of samples, seasons of sampling, post-harvest practices, and hygienic standards applied during the handling, transport, and storage of products, as well as the methods used for isolation and identification of this bacterium. Meat contamination may occur at various stages in preparation including transport, butchering, and cut-up in the kitchen, and the importance of chopping boards as a source of contamination has been reported [31–35].

SE genes were identified in 18 (58.1%) of 31 meat isolates and 42 (51.8%) of 81 clinical isolates. The frequency of enterotoxigenic *S. aureus* in meat samples was higher than that in clinical specimens (*P*> 0.05). According to results, the frequency of SE genes (except *see*) in meat isolates was slightly higher than that in clinical isolates (*P*> 0.05). We found *sea* and *see* genes with higher frequency than others in both meat and clinical samples. Similar to our results, SEA is considered to be the most common cause of food poisoning in Korea and Japan [36]. Furthermore, several studies reported that enterotoxin genes *sea* and *sed* were the most common in staphylococci isolated from food [20, 32, 37]. However, a lower incidence of *sed* (6.4%) was detected in our study. In contrast to our results, *seb* was a prevalent gene in food poisoning cases reported in Taiwan and Japan and *sec* was a major SE gene in isolates from bulk milk in Switzerland and in Korea [36].

It has been known that the *se/sel* genes are carried on mobile genetic elements and most of them contain several *se/sel* genes simultaneously. SE genes are located on plasmids (*sed* and *sej*), phages (*sea*, *see*, and *sep*), and pathogenicity islands on chromosomes (*seb*, *sec*, *seg*, *seh*, *sei*, *sek*, *sel*, *sem*, *sen*, *seo*, and *seq*) [30, 38]. In our study, 55.5% of meat isolates and 38.1% of clinical isolates possessed more than one *se* gene. Seven *se* genotypes were observed in meat isolates, and the most commonly detected were *sea+see* (16.7%) and *sea+seb+see* (11.1%). Nine *se* genotypes were also detected in clinical specimens, and the most frequent combinations were *sea+seb+see* (9.5%) and *sea+ seb* (7.1%), respectively.

## Conclusion

The presence of enterotoxin producing *S. aureus* in clinical and raw meat samples represents a potential health risk. Therefore, intensive and continuous monitoring of potentially pathogenic *S. aureus* is strongly recommended in order to evaluate the human health risk arising from food consumption.

## Data Availability

The datasets will not be available on a publically available website, but it may be possible to provide access to anonymized data.

## Abbreviations

SFP: Staphylococcal food poisoning
SE: Staphylococcal enterotoxin
SCC: Staphylococcal cassette chromosome
ELISA: Enzyme-linked immunosorbent assay
PCR: Polymerase chain reaction
